# Prevalence of the Prefoldin Subunit 5 Gene Deletion in Canine Mammary Tumors

**DOI:** 10.1371/journal.pone.0131280

**Published:** 2015-07-01

**Authors:** Silvia Hennecke, Julia Beck, Kirsten Bornemann-Kolatzki, Stephan Neumann, Hugo Murua Escobar, Ingo Nolte, Susanne Conradine Hammer, Marion Hewicker-Trautwein, Johannes Junginger, Franz-Josef Kaup, Bertram Brenig, Ekkehard Schütz

**Affiliations:** 1 Institute of Veterinary Medicine, Georg-August-University Goettingen, Goettingen, Germany; 2 Chronix Biomedical, Goettingen, Germany; 3 Small Animal Clinic, University of Veterinary Medicine Hannover, Hannover, Germany; 4 Department of Pathology, University of Veterinary Medicine Hannover, Hannover, Germany; 5 German Primate Center, Goettingen, Germany; 6 Hematology Oncology and Palliative Medicine, ClinicIII, University of Rostock, Rostock, Germany; Georgetown University, UNITED STATES

## Abstract

**Background:**

A somatic deletion at the proximal end of canine chromosome 27 (CFA27) was recently reported in 50% of malignant mammary tumors. This region harbours the tumor suppressor gene prefoldin subunit 5 (*PFDN5*) and the deletion correlated with a higher Ki-67 score. *PFDN5* has been described to repress c-MYC and is, therefore, a candidate tumor-suppressor and cancer-driver gene in canine mammary cancer. Aim of this study was to confirm the recurrent deletion in a larger number of tumors.

**Methods:**

Droplet digital PCR for *PFDN5* was performed in DNA from 102 malignant, 40 benign mammary tumors/dysplasias, 11 non-neoplastic mammary tissues and each corresponding genomic DNA from leukocytes. The copy number of *PFDN5* was normalized to a reference amplicon on canine chromosome 32 (CFA32). Z-scores were calculated, based on Gaussian distributed normalized *PFDN5* copy numbers of the leukocyte DNA. Z-scores ≤ -3.0 in tissue were considered as being indicative of the *PFDN5* deletion and called as such. The Ki-67 proliferation index was assessed in a subset of 79 tissue samples by immunohistochemistry.

**Results:**

The deletion was confirmed in 24% of all malignant tumors, detected in only 7.5% of the benign tumors and was not present in any normal mammary tissue sample. The subgroup of solid carcinomas (n = 9) showed the highest frequency of the deletion (67%) and those malignomas without microscopical high fraction of benign tissue (n = 71) had a 32% frequency (p<0.01 vs. benign samples). The Ki-67 score was found to be significantly higher (p<0.05) in the *PFDN5*-deleted group compared to malignant tumors without the deletion.

**Conclusions:**

A somatic deletion of the *PFDN5* gene is recurrently present in canine mammary cancer, supporting a potential role in carcinogenesis. The association of this deletion with higher Ki-67 indicates an increased proliferation rate and thus a link to tumor aggressiveness can be hypothesized. The confirmation of earlier results warrants further studies on *PFDN5* as cancer-driver gene.

## Introduction

Mammary tumors represent the majority of neoplastic diseases in female dogs, constituting over 40% of all female canine tumors [1]. Of these approximately 50% are malignant and in majority being of epithelial origin (simple adenoma/carcinoma). In contrast to human breast cancer 20% of the canine tumors exhibit myoepithelial cell proliferation (complex carcinoma). Canine mammary tumors (CMT) have been proposed as a model for human breast cancer and increasing knowledge about the molecular differences and similarities will support the use of CMT in breast cancer research [2–4]. However, molecular research in canine mammary gland tumors in the recent years seemed to focus primarily on genes with well established roles in human carcinogenesis (for review see: [2]). In order to gain a deeper understanding of molecular processes that can lead to neoplastic transformation of the canine mammary gland a broader spectrum of investigated genes is required.

Recently, our group identified a recurrent deletion of the proximal part of cfa27 by high-throughput sequencing analyses of five canine malignant mammary tumors (MMTs). Within this region *PFDN5* appeared as the most plausible candidate with cancer-driving functional relevance. [5].

The Prefoldin Subunit 5 gene (*PFDN5*) maps to the proximal end of the canine chromosome (cfa) 27 (1,906,133–1,909,613 bp, Broad CanFam 3.1). The encoded protein is part of the prefoldin complex that functions as chaperone and is involved in the posttranslational folding of high-order protein structures such as actin and microtubules [6]. Furthermore, prefoldin and its subunits can localize to the nucleus and act as transcriptional regulators by binding to the DNA or transcription factors [6]. PFDN5 in particular is known to repress the c-MYC transcription factor [7]. Three different c-MYC repression mechanisms are described that are mediated by PFDN5: i) repression of the transcriptional activity by binding to the N-terminal region of c-MYC ii) recruiting of the ubiquitin ligase complex to c-MYC that drives proteasome dependent degradation iii) repression of the Wnt4 expression that leads to the repression of c-MYC expression [6–11]. Therefore, *PFDN5* has the functional potential to act as a tumor suppressor gene. Indeed, a frequent amino acid substitution (A157R) that led to a loss of its c-MYC transcriptional repression activity was found in 50–60% of leukemia/lymphoma cells and in more than 75% of human tongue cancers [8]. Furthermore, tumor-specific alternative splicing variants were detected in thyroid and head/neck cancer [12, 13]. One variant was generated by the retention of intron 2 that generated a premature stop-codon and another detected splice isoform led to the insertion of 101 amino acids [12, 13]. Both splice-variants are expressed at higher levels in tumor as compared to normal tissues or benign lesions [12, 13].

In human breast cancer *PFDN5* was amongst 102 genes for which differential expression in tumor versus normal tissue was detected; 69% of the analysed tumor samples showed a decreased expression [14].

However, *PFDN5* cannot be considered as a well-known established cancer-driver gene, neither in human nor in canine cancers. Nevertheless, significantly higher Ki-67 scores, indicative of a higher proliferative activity, were detected in the tumors with *PFDN5* deletion [5], which can serve as hint for a functional association. Since the sample number in our first explorative study was small the primary goal of this study was to confirm the deletion of PFDN5 in a larger number of canine MMTs compared to benign tissue.

## Material and Methods

### Clinical samples

A total of 153 mammary tissues (CMT n = 142 and non-neoplastic controls n = 11) were investigated, of which 82 were from patients of the Clinic for Small Animals, Institute of Veterinary Medicine, Georg-August-University Göttingen and 71 mammary tissues were sampled at the Clinic for Small Animals, University of Veterinary Medicine Hannover. The CMTs and the non-neoplastic mammary tissues were collected from 127 bitches with a median age of 9 years (range 3–15 years), of which most were intact (16.3% surgically castrated). Cross breeds (n = 35) were the most common breed represented, followed by Cocker spaniels (n = 7), German shepherds (n = 5), Dachshund (n = 5), Golden Retriever (n = 5) and Labrador Retriever (n = 5). A total of 40 different other breeds were included in the study. Considering the composition of the herein analysed samples the median age of occurrence, the affected breeds and distribution of different tumor types in this study were consistent with other reports [1, 15, 16].

Tumors were removed by mastectomy and the diagnosis was confirmed by histopathological evaluation. All tumors and the corresponding peripheral blood mononuclear cells (PBMC) were obtained as part of routine diagnostic procedures with informed consent of the owner. Therefore the study was exempt of ethical approval according to German regulations.

The 153 samples were classified histological using formalin fixed paraffin embedded (FFPE) tumor tissue with haematoxylin and eosin staining according to the published criteria of the World Health Organization [17]. The samples consisted of 102 (67%) MMT (1 non-infiltrating carcinoma, 27 complex carcinomas, 36 simple carcinomas, 5 spindle cell carcinomas, 1 osteosarcoma, 4 carcinosarcomas, 28 carcinomas in benign tumors) and 32 (21%) BMT (17 adenomas, 4 fibroadenomas, 11 benign mixed tumors), 6 (4%) MH, 2 (1%) duct ectasias (DE) and 11 (6%) samples with normal histopathological findings.

In order to separate cancerous and adenomatous parts of 12 carcinomas in benign tumors (6 predominantly carcinomas and 6 predominantly adenomas) the FFPE specimens were macrodissected (malignant and benign parts), by using a 3mm dermal punch and were then examined separately.

### DNA extraction from tissue, blood sampling and FFPE

DNA was extracted from 25mg of homogenized tumor and non-neoplastic tissue and 200μL of the corresponding PBMC using the DNeasy Blood & Tissue Kit (Qiagen, Hilden, Germany). DNA from macrodissected carcinoma in benign tumor FFPE tissues was extracted using the Gene Read DNA FFPE Kit (Qiagen, Hilden, Germany) following the manufacturers instructions.

### Droplet Digital PCR assays

A ddPCR assay was designed to evaluate a deletion at the proximal end of chromosome CFA27 in the tumor genomes. The targeted region harbours the annotated *PFDN5* gene locus (GeneID: 607260; location CFA27: 1,906,133–1,909,613bp on CanFam3.1 reference genome; NCBI; NW_006609.3). Primers and probes were designed using the Primer3 program (http://Frodo.wi.mit.edu/primer3/). All sequences were cross-checked against the canine reference genome to ensure unambiguous results. The sequences of the primers and probe were: CFA27.F: 5´-GCCTTAGAACTATACCTATACATTCCG-3´, 5´-CFA27.R: GTAGCATATGTCATTTTCTCCATAAGG-3´ and CFA27-Probe: 5´-6-Fam-ACCGACTTCTCTGTTCCTTCCCATTC-BHQ1-3´ yielding an 80bp amplified fragment (CFA27:1,908,080–1,908,159; Broad CanFam3.1) in the intronic region. A 79bp fragment on CFA32 (location CFA32: 2,342,421–2,342,499; Broad CanFam3.1) served as control amplicon; this region had equal copy-numbers in PBMC/tumor pairs of the included dogs. The sequences of the primers and probe for CFA32 were: CFA32.F: 5´-AAAAGCCTCCAATCCCCGAG-3´, CFA32.R: 5´-CCTGACAGAAAAAGCAGCCC-3´ and CFA32-Probe: 5´-HEX-CTCCGTGACAAGTCAAGCTCAATAGCCT-BHQ1-3´.

1μg of genomic DNA of the tumors and corresponding PBMCs was digested with 5 U *Apo*I restriction endonuclease in a total reaction volume of 25μL. 60ng digested DNA, 10μL 2x ddPCR Master Mix (Bio-Rad, Munich, Germany), 900nmol/L of each primer and 250nmol/L of hydrolysis probes were supplemented with nuclease- and protease free water to a final volume of 20μL. Approximately 23,000 droplets per reaction were generated using the QX200 Droplet Generator (Bio-Rad, Munich, Germany) and PCR amplification was performed in a thermal cycler (Biometra, Göttingen, Germany) at 95°C for 10min followed by 50 cycles at 95°C for 30sec and 60°C for 1min, 98°C for 10min and 12°C hold.

The droplets were analysed using the QX200 Droplet Reader (Bio-Rad, Munich, Germany). On average ddPCR yielded 11,252 (SD:1,505) detected usable droplets in PBMC DNA and 11,653 (SD:1,471) detected droplets in the tissue DNA. Using the QuantaSoft software the concentration of the respective template and the relative standard uncertainty was calculated based on the Poisson distribution. The obtained copy number of the target amplicon in the *PFDN5* gene locus was calculated relative to the CFA32 reference amplicon (diploid).

### Immunohistochemistry

Immunohistochemistry was performed on a subset of 79 paraffin-embedded samples using the avidin-biotin complex (ABC) method (VECTASTAIN Elite ABC kit, Vector Laboratories, Burlingame, California, USA) and 3,3’-diaminobenzidine (DAB, Sigma-Aldrich, Munich, Germany) to produce a brown colour reaction as described elsewhere [18].

Briefly, antigen retrieval was performed in citrate buffer (pH 6.0) for 20min at 95°C followed by blocking of endogenous peroxidase activity with 0.5% hydrogen peroxide in 70% ethanol for 30min. Subsequent to the application of normal goat serum (20% in PBS, 30 min), primary mouse anti-Ki-67 antibodies (1:100 in PBS containing 1.0% BSA: clone MIB-1, Dako, Hamburg, Germany) were added and incubated over night at 4°C. Biotinylated goat anti-mouse IgG secondary antibodies (1:200 in PBS, Vector Laboratories) were applied for 30min at room temperature. Sections were counter-stained with Mayer’s hematoxylin solution. Specimens from normal canine lymph node were used as positive control. Primary antibodies were replaced by ascites fluid from non-immunized BALB/cJ mice (Cedarlane, Ontario, Canada) in equal concentrations for negative controls.

Sections were photographed at 200x magnification in five randomly selected areas of the highest nuclear labelling. A total of at least 350 proliferating and non-proliferating cells were counted manually to calculate the percentage of Ki-67 expressing cells.

### Statistical analysis

For the evaluation of the frequency of the deletion in the tumor samples, the obtained copy-numbers were converted into z-scores, which were based on the average copy numbers measured in the PBMCs of all animals (μ = 2.02, SD = 0.09). For comparisons of *PFDN5* deletion a T-test assuming equal variance was used. A deviation of z ≤ -3 was regarded as significant aberration, and differences in the group frequencies were compared using Fisher’s exact test (one-tailed). Since not Gaussian distributed (KS-test), the Ki-67 scores were compared using the Mann-Whitney U-test. Samples from BMT, MH, DE and normal tissue where grouped as being of benign origin.

## Results

Overall, the relative copy numbers of *PFDN5* in malignant tumors (n = 102) was significantly lower (p<0.05) compared to benign tissue (n = 51), while, no significant difference was present in PBMC genomic DNA for the same groups (p = 0.16). If those tumors with high amount of benign tissue (carcinoma in benign tumors) and the carcinoma in situ were omitted from this analysis, the remaining 74 cases showed a highly significant deviation from benign controls (p<0.01).

Hence, by using a z-score delimiter of -3.0 the frequency of the *PFDN5* deletion was higher in malignancies (n = 102), yielding an odds ratio of 4.9 (95% Cl: 1.4–17.2, p = 0.004 Fisher’s exact test).

At that delimiter of z = -3.0, the deletion was detected in 26% of complex carcinomas (7|27), in 21% of tubulopapillary carcinomas (3|14) and in 67% of solid carcinomas (6|9). Furthermore the deletion was detected in one simple carcinoma (1|10), one anaplastic carcinoma (1|3), one spindle cell carcinoma (1|5), one osteosarcoma (1|1) and in one carcinosarcoma (1|4). In the most frequently occurring carcinoma/sarcoma in benign tumors (3|28) the deletion was detectable in 11%. In 9% (3|32) of the BMTs the deletion was detected, which were one complex adenoma and two benign mixed tumors. In the 19 non-neoplastic mammary tissues, including the six MH and the two DE the deletion was not detected (Table 1).

**Table 1:** *PFDN5* deletion in different tumor groups.

Malignant tumor	n	Del Z-Score	%
Non-infiltrating (in situ) carcinoma	1	0	0
Complex carcinoma	27	7	26
Simple carcinoma	10	1	10
Tubulopapillary carcinoma	14	3	21
Solid carcinoma	9	6	67
Anaplastic carcinoma	3	1	33
Spindle cell carcinoma	5	1	20
Osteosarcoma	1	1	100
Carcinosarcoma	4	1	25
Carcinoma or sarcoma in benign tumor	28	3	11
**Total**	102	24	24
**Benign tumor**			
Simple adenoma	3	0	0
Complex adenoma	14	1	7
Fibroadenoma	4	0	0
Benign mixed tumor	11	2	18
**Total**	32	3	9
**Other non-malignant**			
Hyperplasia (ductal/lobular)	6	0	0
Duct ectasia	2	0	0
Normal adjacent tissue	11	0	0
**Total**	19	0	0
**All non-malignant**	51	3	6

In samples that were histopathologically categorized as malignancy within benign tumors, the content of DNA from malignant cells is usually low in total tissue, which may result in failure to detect the deletion, even if present in malignant cells. To prove this, in a set of 6 tumors, the malignant and the benign parts were macrodissected from FFPE samples, which were scored negative of for the *PFDN5* deletion, when homogenized total tissue was used. In contrast to the results in tissue, the deletion was found in three of the predominantly malignant parts (50%) but not in the areas, which were defined as predominantly adenomas (0|6).

Immunolabelling with Ki-67 antibodies was performed in a subset of 100 tissue samples and revealed a clear nuclear labelling that showed a variable intensity. Due to the low content of malignant cells in the so called carcinomas in benign tumors (n = 21), the KI-67 was reduced, congruent with the low abundance of the PFDN5 deletion in the whole tissue of that tumor type. This type was therefore excluded from statistical analysis to avoid a bias in favour of an association between KI-67 and the somatic deletion. The percentage of Ki-67 expressing cells was evaluated in 79 tumor samples (MMT n = 50; BMT n = 21; MH n = 6 and DE n = 2) and ranged between 4% and 81%. Ki-67 expression was significantly higher (p = 0.0001; Mann-Whitney U-test) in MMT (median: 27%, 25^th^-75^th^ percentile: 21.3%-33%) compared to benign neoplasms (median: 15%, 25^th^-75^th^ percentile: 13%-24%) (Table 2).

**Table 2:** Ki-67 score and SD in different tumor groups.

Malignant Mammary Tumors	n	median (%)	min (%)	max (%)
In situ carcinoma	1	26.6		
Complex carcinoma	17	23.7	4.1	42.9
Simple carcinoma	2	47.1	13.5	80.7
Tubulopapillary carcinoma	10	26.7	11.1	48.9
Solid carcinoma	8	33.4	21.8	57.1
Anaplastic carcinoma	2	25.4	23.7	27.1
Spindle cell carcinoma	5	27.7	17.6	30.9
Osteosarcoma	1	22.6		
Carcinosarcoma	4	29.3	22.3	46
**Total**	50	27	4	81
**Non-malignant Mammary Tissue**				
Simple adenoma	2	14.9	14.3	15.4
Complex adenoma	11	15.4	7.4	49.4
Fibroadenoma	3	29.4	22.6	34
Benign mixed tumor	5	14	6.9	27.1
Duct ectasia	2	15.2	15	15.4
Hyperplasia	6	15.6	4.0	33
**Total**	29	15	4	49

**Fig 1:**
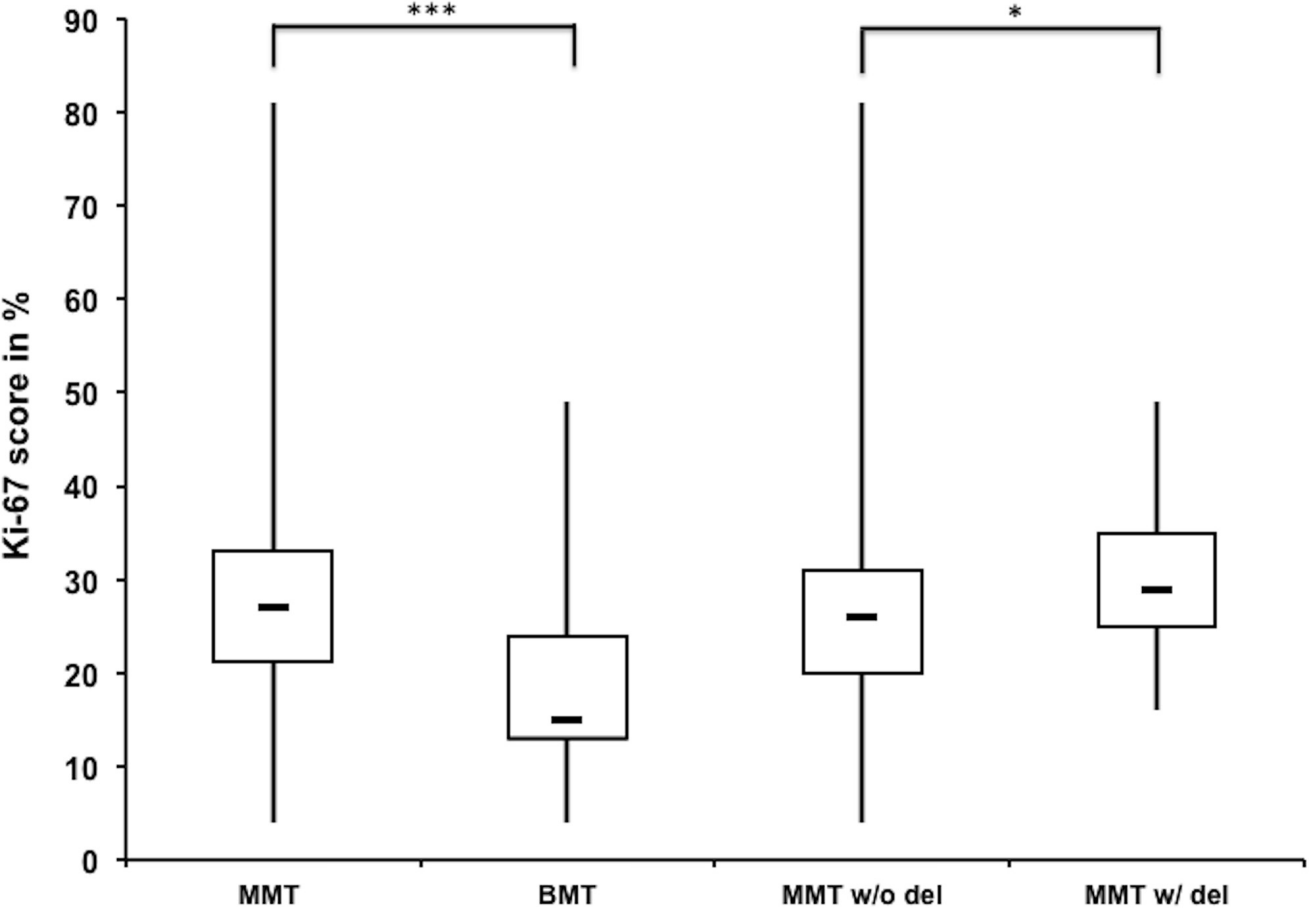
Box plot of Ki-67 scores. Boxplots of Ki-67 score in all malignant mammary tumors (MMT), all benign mammary tumors (BMT) (*** p<0.0001), in malignant mammary tumors with (w/) and without (w/o) the *PFDN5* deletion and without the carcinoma in benign tumor (* p<0.05)

In the MMTs a significant higher Ki-67 expression between the *PFDN5*-deleted (median: 29%, 25^th^-75^th^ percentile: 25%-35%) and non-deleted group (median: 26%, 25^th^-75^th^ percentile: 20%-31%) was present (p = 0.05; Mann-Whitney U-test; Fig 1).

## Discussion

This study was intended to validate our earlier finding [5] on the presence of a recurrent somatic deletion of the *PFDN5* gene on CFA27. The results confirm that this somatic deletion is present in canine MMT and represents one potential new tumor marker. Furthermore, the somatic presence of the deletion in 23% of the analysed malignant tumors supports *PFDN5* as being a potential tumor driver gene. Cancer drivers are defined as genes that when somatically altered contribute to the development of cancer [19, 20]. It is thought that several different mutations are required to drive oncogenesis, the actual number, however, can only be estimated and is variable between different cancers [20]. Cancer driver genes are commonly thought to be altered at a certain level of recurrence [20]. An amplification of the c-MYC oncogene for example is found in 15.7% of human breast cancers [21].

In the current study the highest deletion frequency (67%) of canine *PFDN5* was detected in the group of solid carcinomas followed by 26% in complex carcinomas. On the other hand, only 10% of the analysed simple carcinomas carried the deletion. These differences might be indicative of different carcinogenic mechanisms that give rise to the different carcinoma types, as recently suggested [3].

The very low percentage of detected deletions in the special types of carcinoma or sarcoma in benign tumors is most likely due to the "dilution” of the often low numbers of malignant cells by the benign fraction of the tumor. In these tumors foci of malignant-appearing cells or distinct nodules of malignant cells reside within complex adenomas or benign tumors [22]. Our results in macrodissected malignant areas, where the deletion was found in 3 of 6 samples that were not detectable when homogenized total tissue was used, confirmed such a dilution effect of the DNA from malignant cells by the benign fraction.

Interestingly, the deletion was also found in a few BMTs, though they did not show histopathological evidence of malignancy at the time of surgery. However, those three cases were in complex and mixed tumors, where the histopathological evaluation is extremely difficult, a recent survey unveiled a considerable false negative rate [23], furthermore focal differences between the section used for microscopy and for molecular analyses can never be excluded.

On the other hand, benign mammary tumors (BMT) have been considered as precancerous lesions and carry an increased risk of malignant transformation [24]. In this line it could also be hypothesized that a deletion of the c-Myc repressor gene *PFDN5* may be a risk indicator for precancerosis.

Further indication for the proposed role of *PFDN5* as tumor-suppressor is the detected association between CFA27 deletion and higher Ki-67 scores. Ki-67 antigen is a non-histone protein, which is only expressed in proliferating cells and enabling to determine the growth fraction of a cell population [25] and cell proliferation in mammalian tumors e.g. humans and dogs [4]. In CMT increased cell proliferation may serve as an indicator of malignancy since Ki-67 was reported to be significantly different between BMT and MMT [2]. High Ki-67 scores were associated with increased tumor grade, larger tumor size, lymph node involvement and metastasis and the survival time was significantly decreased for bitches with high Ki-67 score tumor [2]. Even though a statistical significant difference between Ki-67 scores of malignant and benign tumors was seen in our sample set, the large overlap (cf. Fig 1) seems to compromise a general use as prognostic marker in dogs. Similarly, Ki-67 scores in malignant tumors are significantly higher in those with the deletion compared to tumors without the deletion, but again both groups largely overlap.

Due to the location of *PFDN5* close to the centromere it can not be excluded that its frequent deletion in canine mammary tumors is solely related to its position. Centric fusions that result from reciprocal translocation appear frequently in canine tumor cells and are thought to occur randomly and with greater frequency at later stages of tumor progression [26]. Reciprocal translocations often lead to large deletions at the breakpoints [27]. Therefore, it is possible that the recurrent deletion of PFDN5 in canine tumors is attributable to its position rather than to its function during carcinogenesis. Nevertheless, the fact that such a somatic telomeric deletion was not found for other chromosomes and that the PFDN5 deletion has a substantial frequency in malignant tumors makes a functional role well conceivable.

## Conclusion

The present study confirms and extends our previous findings of the abundance of a deletion of the proximal part of CFA27 harbouring the *PFDN5* gene in canine mammary tumors. This confirmation warrants further studies including the assessment of biochemical and in vitro data that evidently prove the functional link to *PFDN5* and oncogenic transformation. With the current knowledge, the question as to whether the observed *PFDN5* deletion is a primary or secondary driver event during tumorgenesis cannot be answered.
